# Monkeypox in Patient Immunized with ACAM2000 Smallpox Vaccine During 2022 Outbreak

**DOI:** 10.3201/eid2811.221215

**Published:** 2022-11

**Authors:** Matthew Turner, Jeremy Mandia, Case Keltner, Robert Haynes, Paul Faestel, Luke Mease

**Affiliations:** Madigan Army Medical Center, Tacoma, Washington, USA

**Keywords:** monkeypox, monkeypox virus, viruses, acute infection, immunization, vaccination, ACAM200 smallpox vaccine, sexually transmitted infections, vaccine-preventable diseases, men who have sex with men, vaccines, United States

## Abstract

We report a case of monkeypox in the United States in a patient who had been vaccinated with ACAM2000 smallpox vaccine 8 years earlier. Despite his vaccination status, he still contracted disease. He showed prodromal symptoms preceding development of painless penile lesions that later coalesced.

In the summer of 2022, the Centers for Disease Control and Prevention initiated an emergency response because of a national outbreak of infection with monkeypox viru*s*. On June 28, 2022, the US Department of Health and Human Services announced a national monkeypox vaccination strategy to contain the pandemic (*1*).

We report a patient in Washington, USA, who contracted monkeypox despite being successfully immunized against smallpox with the ACAM2000 smallpox vaccine (https://www.sanofi.com) 8 years earlier. We pose major questions regarding the efficacy of ACAM2000 vaccine amidst ongoing shortages of the JYNNEOS (https://www.bavarian-nordic.com) 2-dose monkeypox vaccine.

The patient was a previously healthy 34-year-old man who had sex with men came to a walk-in sexually transmitted infections clinic because of a 4-day history of malaise, fatigue, and headache and a 2-day history of 4 painless penile lesions. The patient had sought evaluation at a local emergency department 2 days before he visited the clinic. Results for testing performed in the emergency department were negative for *Neisseria gonorrhea*, *Chlamydia trachomatis*, and herpes simplex virus. His constitutional symptoms improved over the next 2 days. However, his penile ulcers progressed into white papular lesions, prompting him to seek reevaluation.

The patient had a medical history of noncomplicated *N. gonorrhea* infection and syphilis in 2017 that resolved after treatment. He had no history of HIV infection or other immunocompromising condition documented in his military health records. He was previously prescribed daily emtricitabine/tenovir as preexposure prophylaxis for HIV, but he self-discontinued a year before he sought care. In the previous 90 days, he reported penetrative anal and receptive oral sexual intercourse with 13–14 new partners, denying any condom use. His last sexual intercourse was 11 days before he sought care, when he engaged in unprotected anal-insertive sex with a single anonymous partner at a local Pride event. Because of his military service, he was vaccinated against smallpox with ACAM2000 smallpox vaccine in March 2014, with documented vaccine take. He denied recent travel outside Washington or exposure to sick contacts.

On examination, the patient had 4 ulcerated penile lesions that had consolidated into a 3-cm patch present on the foreskin, 2 days after constitutional symptoms developed (Figure, panel A). The lesions were nontender to palpation, and no discharge was present. A tender 3-cm right inguinal lymph node was present. A vaccination scar was noted on his right deltoid, but the remainder of the examination was unremarkable.

**Figure:**
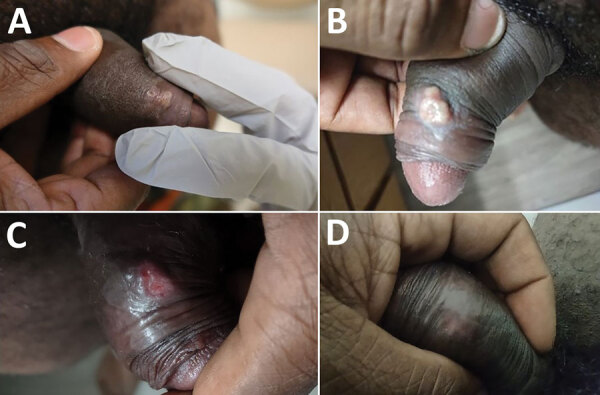
Evolution of penile lesions in patient who had monkeypox and was immunized with ACAM2000 smallpox vaccine during 2022 monkeypox outbreak, United States. A) Two days after constitutional symptoms developed; B) evolution of rash showing coalescence and development of a pustular appearance 6 days after onset of constitutional symptoms; C) ulceration of lesion on day 16; D) dissipation of lesion without residual scarring.

Given the condition of the patient and his sexual history in the setting of an emerging monkeypox outbreak throughout the United States, a nonvariola orthopoxviru*s* PCR was conducted, and the result was positive. Subsequent confirmatory testing by the Centers for Disease Control and Prevention later identified the infection as the clade II strain (formerly West African clade). Additional serum studies, including HIV-1/2 antigen and antibody screening, syphilis screening, and hepatitis C virus screening, showed negative results.

Clinically the patient did well, only requiring supportive care with oral acetaminophen for constitutional symptoms, which resolved 10 days after symptom onset. The rash continued to evolve, coalesced, and developed a pustular appearance 6 days after onset of constitutional symptoms (Figure, panel B). The lesion ulcerated on day 16 (Figure, panel C), and ultimately dissipated without residual scarring (Figure, panel D).

Since the discontinuation of the global smallpox vaccination campaign after eradication of the disease in 1980, monkeypox is the primary circulating orthopoxvirus of public health concern. The ACAM2000 live vaccinia virus vaccine that this patient received in 2014 has been shown to provide protection against monkeypox (*2*,*3*). Earlier studies have reported that among persons vaccinated, monkeypox cases tend to be mild in number of lesions and prodromal symptoms (*4*–*6*). A study in 1988 reported that smallpox vaccination offered ≈85% protection against monkeypox (*4*,*7*). A study in 2008 reported that ACAM2000 vaccine fully protected cynomolgus monkeys after a lethal dose of monkeypox virus; 1 vaccinated animal had a minor rash at the site of inoculation (*8*), which is largely consistent with the manifestations and clinical course of this patient.

Although the mild manifestations in this patient might be attributable to his vaccination against smallpox, it did not prevent infection. The JYNNEOS vaccine is a nonreplicating vaccine product that has a Food and Drug Administration indication to protect against smallpox and monkeypox (*9*). However, the JYNNEOS and ACAM2000 vaccines present disparate challenges. Specifically, the JYNNEOS vaccine is administered as a 2-dose regimen that shows a mild side effect profile, and the ACAM2000 vaccine is a single inoculation that can induce severe adverse effects. However, because of persistent JYNNEOS shortages hampering preexposure and postexposure prophylaxis efforts, vaccination with ACAM2000 might be an option in locales that urgently need immunizations protective against monkeypox. The efficacy of either vaccine in the current outbreak remains unknown.

Although vaccination is foundational for preventing infectious disease, this case highlights that vaccination alone does not guarantee immunity from monkeypox. Public health leaders should taper expectations that vaccination alone will end the outbreak. Vaccine should complement, not replace, public health campaigns that aim to minimize high-risk health behaviors.
